# Feasibility Study of a Piezo Actuator as a Potential Standard in Calibration for Roundness Instruments

**DOI:** 10.3390/s22239312

**Published:** 2022-11-29

**Authors:** Anna Trych-Wildner, Krzysztof Wildner, Piotr Sosinowski

**Affiliations:** 1 Central Office of Measures, Time and Length Department, Precise Geometric Measurements Laboratory, Elektoralna 2, 00-139 Warsaw, Poland; 2 Warsaw University of Technology, Faculty of Mechatronics, Institute of Metrology and Biomedical Engineering, A. Boboli 8, 02-525 Warsaw, Poland

**Keywords:** calibration, roundness, step height standard, piezo actuator, interferometer

## Abstract

The paper presents experimental data that show the possible application of a piezo actuator in the role of calibration standard that can serve as an alternative route to currently available methods like gauge blocks or flick standards. First, the experimental setups for interferometric and roundness instruments measurements were described. Next, the experiments using an interferometer for calibration of the piezo actuator were shown. Finally, the application of a piezo actuator to calibrate the roundness instrument, to state the correction factor for the roundness probe, and to relate it to the unit of metre, assuring the traceability of future measurements was performed. Detailed procedures of simulating grooves and then processing the data were described and the calibration curve was obtained using regression analysis. The estimation of uncertainty provided by different factors during the measurements was given to fulfil as closely as possible real calibration procedures taken up in the measurement laboratories. Finally, the limitations of the presented procedures were presented and discussed.

## 1. Introduction

Touching probes are one of the main physical sensors that are widely used in measuring instruments [1,2,3,4,5]. Their performance strongly depends on the proper design and manufacturing for the specific purpose [4,5,6]. However, once they are deployed for the task, they must be maintained, characterised, and properly calibrated to fulfil their function accurately.

Calibration of the instruments is the crucial operation to ensure that the instrument performs its task accurately and to provide comparability between instruments and results [7,8]. It also provides an adjustment to the measuring devices and ensures traceability of the measurements [9,10,11]. Depending on the instrument’s complexity, the calibration may consist of one or more tasks to ensure the accuracy of the measurements.

In the calibration of the roundness instruments, there are many steps and methods that can be applied [12,13]. In this paper, the authors focus on the feasibility of one of the operations that potentially can be applied as an alternative route to calibrate, check and adjust the amplification factor of the touching probe in roundness instruments.

Unusually, to check and accordingly adjust the probe during the calibration procedure, as a part of the calibration of the whole instrument or as a part of routinely checking the device’s amplification factor or so-called gain factor, one of a few methods is applied. For example, in Taylor Hobson roundness instruments the testing probe is designed in such a way that at the end of the long arm (around 100 mm) the change in the stylus movement might be as small as 0.001 μm. Thus, the movement of the armature is very small and the output from the bridge circuit has to undergo considerable amplification to give an output of sufficient amplitude for conversion to digital data [14]. Typically, to perform the checking procedure of this parameter of the system, the set of gauge blocks with known height is applied [15,16]. This is static measurement, meaning with no rotation of the table and far from real measuring conditions where the instrument is being deployed to measure circular artefacts. More importantly, the step of the wrung gauge blocks strongly relies on the operator’s experience and skills in the operation of blocks wringing for this purpose [17,18].

**Figure 1:**
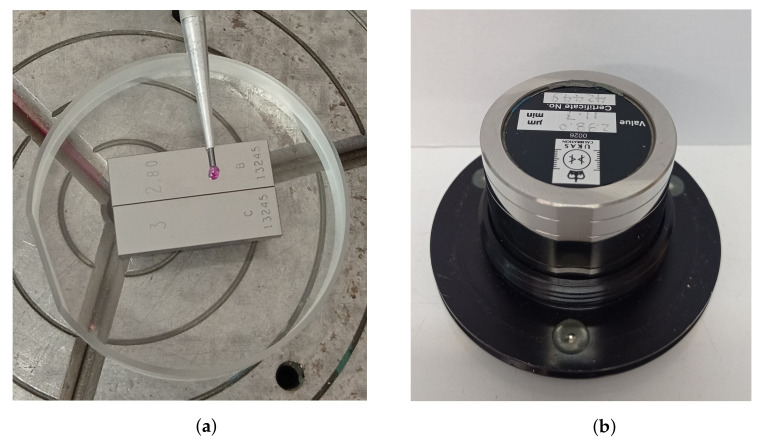
(**a**) Height standard made from two gauge blocks wrung to the optical flat with a visible stylus probe on one of the blocks; (**b**) Flick standard with one surface step.

The height standard used during calibration is a combination of gauge blocks wrung to the optical flat—an example of the step of a nominal value 200 μm is presented in Figure 1a.

The other alternative method for verification of the gain factor is the application of the flick standard [19,20,21]. This is based on a similar concept as wrung gauge blocks but there is a step on the cylinder surface (flat surface built within the artefact)—Figure 1b. This approach is similar to one or sometimes two steps built on the optical flat using gauge blocks. More importantly, a flick standard is usually calibrated using a roundness instrument, i.e., the same kind of instrument and method for which flick standards subsequently serve as calibration standards [22]. Furthermore, in order to ensure a correct traceability chain, the instrument used for calibration must satisfy higher requirements, which means that it must be fully characterised and calibrated by an independent procedure [22]. However, flick standards are mainly used to verify the dynamic response of the instrument [13,23,24,25], unlike wrung gauge blocks where they are mainly used in static measurements, thus, making these methods slightly different.

Nonetheless, both approaches with a flick standard or gauge blocks are limited to only one or sometimes two heights available on a surface. That is why, considering different route that is able to simulate more steps is worth investigating. Up till now, some other techniques have been researched, for example, designing a multi-wave standard [13] or developing a piezo vibrating platform that was prepared for probe dynamic performance calibration [25]. The concept of the more versatile calibration of touching probes with piezo devices was being investigated where utilising so-called moving tables have been appearing, for example, studying the problem even with nanometric accuracy [26,27], analysing the calibration of different systems and different instruments such as roughness and roundness testers or even Atomic Force Microscopes [28] or designing low-cost platforms to measure the frequency response of surface texture measuring instruments [29]. Different approaches for the calibration of measuring instruments show that piezo actuators are being considered as an alternative route to physical artefacts and standards. In this respect, the authors propose a step-by-step route to calibrate a roundness instrument probe, which assumes simulating with a piezo actuator various steps as they could be found on the real physical flick standard or built from gauge blocks. The procedure consists of the calibration of the piezo actuator, which is then transferred to the roundness instrument for calibration. Such an approach will not eliminate well-established methods used so far but can be treated as a complementary and more flexible method, meeting the user’s specific needs. The proposed procedure can be regarded as a generalisation of the gauge blocks method. The user can theoretically choose any size of the step/groove and is not limited to gauge block sizes. The methods such as presented in [24,26,27] tend to focus on dynamic calibration of the probes which can be regarded as similar to the calibration with flick standard. Some methods [25], although ultra-precise, because of their uniqueness and complexity are applicable in the highest standard institutions (such as National Metrology Institutes—NMIs), not necessarily as the simple method to be transferred to industry. The approach presented in this paper is intended for more general use. It can be applied with various piezo actuators currently available for the end user, which makes it relatively affordable.

## 2. Materials and Methods

### 2.1. Measurement Setup

The experimental setup consisted of two separate sets: one for the interferometric measurements and the second for the measurements with the roundness instrument. The piezo actuator was transferable between them.

**Figure 2:**
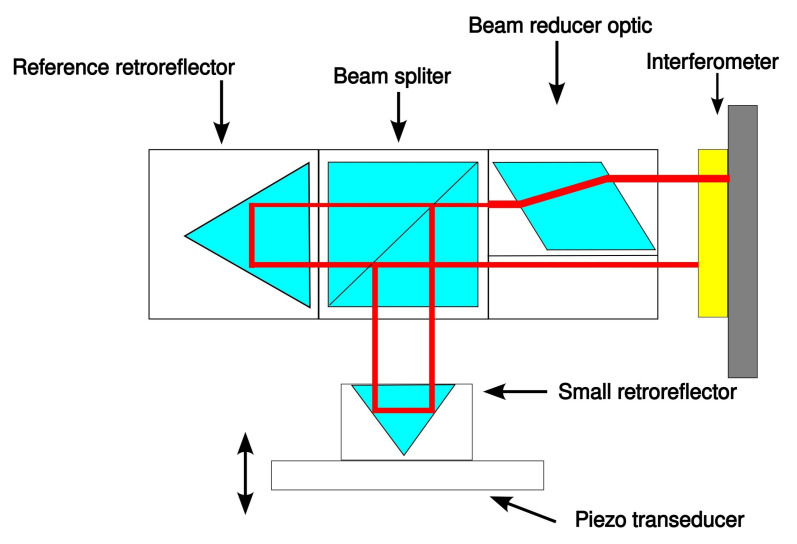
Schematic view of the optical path for the measurements with the XL-80 interferometer system (Renishaw).

**Figure 3:**
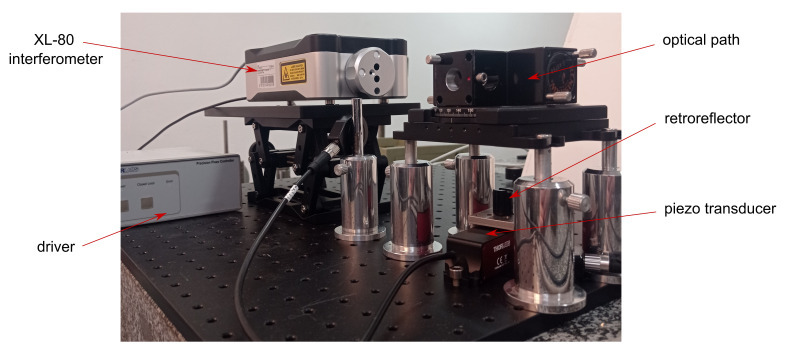
LPS710M (Thorlabs) piezo actuator during calibration with the XL-80 interferometer system (Renishaw). The acquisition was made with Laser XL system’s software (Renishaw), dynamic linear option.

**Figure 4:**
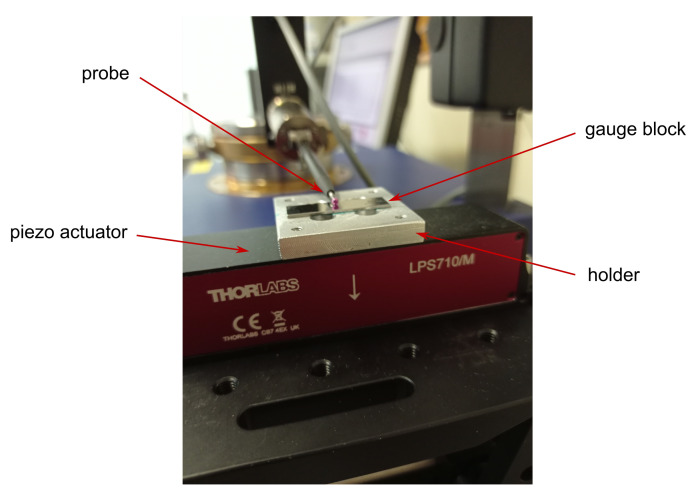
Thorlabs piezo actuator LPS710M with the touching probe of the Taylor Hobson 130 roundness instrument put on the gauge block surface.

(a) Interferometer
  To perform the experiments the XL-80 interferometer system (Renishaw, Wotton-under-Edge, UK) was used (accuracy: ±0.5 ppm, linear resolution: 1 nm) along with the Renishaw small optics kit (A-8003-3244), which includes a lightweight retroreflector. The schematic view of the setup is presented in Figure 2 and the photograph of the whole set with piezo actuator is in Figure 3.
  The interferometer was selected to work in the linear dynamic mode to collect the data while the position of the piezo actuator was changing dynamically. A relatively high sampling rate (50 kS/s) was chosen, as the acquired data were aimed to be used to characterise the response of the piezo actuator, besides the calibration of the device itself.
(b) Roundness instrument
  The Taylor Hobson 130 (Leicester, UK) roundness measurement instrument was used as a device to be calibrated with a standard based on a piezo actuator. The resolution declared by the manufacturer, used when component deviations are less than 0.40 mm, is 6 nm. The Ultra^®^ (Taylor Hobson) software was used to collect the data. Further details can be found in [30]. The configuration of the device, while carrying out the experiments, was different than usually used when working with roundness-measurement instruments. The piezo actuator was located outside the turntable and the probe of the roundness instrument was oriented horizontally, using possible movements of the arm and the column of the instrument. The tip of the probe was touching the surface of the gauge block mounted to the piezo actuator (Figure 4).
(c) Piezo actuator
  For the experiments, the LPS710M (Thorlabs, Newton, NJ, USA) piezo actuator was used (Figure 4). All the tests were performed in a closed-loop configuration available for the actuator. For the closed-loop operation key specifications declared by the manufacturer are as follows: resolution: 6 nm, accuracy: ±0.06%, bidirectional repeatability: ±0.03%, angular error: ±40 μrad, maximum load: 300 g, displacement range: 800 μm—the detailed specification can be found in [31]. A small holder (custom-made) was screwed to the top surface of the actuator and the gauge block (to put the touching probe on the clean, flat, with-little-roughness surface) or retroreflector was fixed to the holder while working with the roundness measurement machine or the interferometer, respectively. The movement of the piezo actuator was programmed with Kinesis^®^ (Thorlabs) software. The actuator was driven by a dedicated Thorlabs PPC001 Piezo Controller. Key parameters of the driver—drive voltage stability: 100 ppm over 24 h, drive voltage noise: <0.5 mV RMS (20 Hz–100 kHz)—further details can be found in [31].

### 2.2. Methodology

The procedure consisted of two subsequent parts. First, the laser interferometer XL-80 was used to calibrate the LPS710M piezo actuator. This actuator was intended to simulate a set of real standards/artefacts with grooves present at their surface. The calibration was performed for six grooves depths: 0.24 μm, 0.75 μm, 2.4 μm, 7.5 μm, 24 μm and 75 μm. The grooves generated for the experiments were performed according to the sequence prepared with the dedicated Kinesis^®^ software (Thorlabs).

Next, the piezo actuator, calibrated in such a way, was used as a reference standard in the calibration procedure of the Taylor Hobson 130 roundness measurement machine. The calibration was performed using the same grooves’ depths, as mentioned above.

### 2.3. Data Collecting Procedure

**Figure 5:**
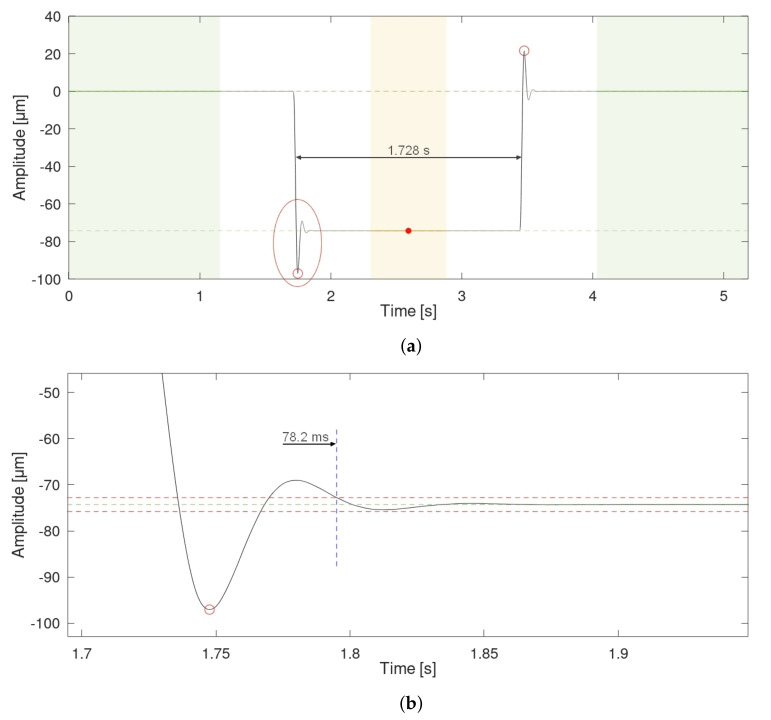
(**a**) Example of ringing oscillations observed in the simulated groove of 75 μm. The green dashed lines represent the model fitted according to ISO 5436-1:2000 standard. These lines are extrapolated along the time axis for visual guidance. The red ellipse indicates the zoomed-in area shown below. Small red circles indicate peaks of the oscillations: 22.8 μm for the falling edge and 21.9 μm for the rising edge respectively. Green-shaded areas are placed over external measurement lengths and the orange-shaded area is placed over the central measurement length. The red dot indicates the middle of the central length; (**b**) Zoomed-in oscillations at the falling edge of the groove. Red horizontal lines represent 2% deviation from the target depth. The blue vertical line represents the end of the settling time. The settling time equals 78.2 ms.

All the measurements were performed in the Precise Geometric Measurements Laboratory, Central Office of Measures, Warsaw, Poland under stable environmental conditions. The piezo actuator was driven to simulate grooves of the required depth repeatedly. To obtain the piezo actuator movement a square excitation was used, as it is straightforward to implement. However, such excitation tends to result in damped ringing oscillations that might affect the measurements (Figure 5).

To mitigate the influence of such oscillations on the results, changes in the actuator position were held for at least 10 folds the settling time of the actuator. The settling time was expressed as the time elapsed from the moment the actuator reached 2% of the required groove’s depth to the moment from which oscillations around the target position were lower than 2% of the required depth. The settling time was approximately 75–80 ms, regardless of the target depth. After adding some additional margin, we decided on grooves to be a minimum of 1 s wide.

**Figure 6:**
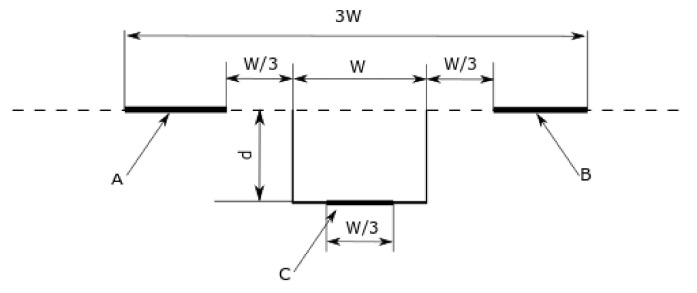
Typical schematic view for calculation of the groove’s depth according to the ISO 5436-1:2000 standard; *W*—the width of the groove, *d*—the depth of the groove, *C*—the line segment in the middle of the groove, *A* and *B*—the line segments on the surface of the standard.

To meet the International Organization for Standardization (ISO) standard for geometrical product specifications requirements [32], the measurement lengths need to be equal $\frac{1}{3}$ and $\frac{2}{3}$ the width of the groove for the middle (Figure 6, line segment C) and the external (Figure 6, line segments A,B) measurement lengths, respectively. $\frac{1}{3}$ the width of the groove needs to be rejected in both directions around the groove edges (Figure 6). Thus, while generating a series of grooves the distance between subsequent grooves should be at least equal to $1\frac{1}{3}$ the width of the groove.

The piezo actuator used during measurements was controlled by its native software. However, it did not hold time dependencies properly. The inaccuracy of the width of the grooves was at the level of 20% or more. Thus, the measurement lengths had to be calculated for each groove separately, based on the actual groove’s width. The minimum required distance between the particular groove and the previous groove was chosen to be equal to the width of the groove increased by the $\frac{1}{3}$ width of the previous groove and additional $\frac{1}{3}$ width of the groove being measured. The last component served as a margin for the edge-seeking and data-splitting algorithm. In our measurements, we used a larger distance between subsequent grooves than the minimum required, i.e., the distance was equal to 2 and 4 times the assumed groove’s width, for the interferometric and roundness machine measurements, respectively. Nevertheless, this distance was always verified during the validation of the acquired data.

(a) Interferometer data
  The calibration of the piezo actuator, performed with an XL-80 interferometer (Figure 2 and Figure 3) involved measurements of six grooves’ depths (as mentioned above). For each depth, two 40 s acquisitions were performed with a sampling rate of 50 kS/s (Laser XL system’s software, dynamic linear option, Renishaw). The initial position of the actuator was 100 μm, which is $\frac{1}{8}$ of the device range. The actuator position was decreased for 1.5 s by a distance equal to the desired groove depth, then it returned to the initial position and after 3 s of break, the pattern was repeated. This resulted in 8 repetitions per single acquisition, 16 repetitions per each depth (two measurement series). Original data were stored within a binary block inside 12 Renishaw ‘.rtx’ files (six depths, two files per each depth), represented as double-precision floating-point numbers.
(b) Taylor Hobson data
  The calibration of the Taylor Hobson 130 roundness measurement machine, based on the piezo actuator, involved the simulation of the same grooves’ depths as in the case of calibration of the actuator with the XL-80 interferometer. Data were collected in the same way as when performing typical roundness measurements, but with a different arm position and with the reference standard located out of the turntable. The acquisition time was determined by the speed of the machine’s turntable, which was 6 RPM. One full revolution of the turntable took 10 s and that was the acquisition time. The sampling frequency was equal to 360 S/s. The measurements were performed without an external synchronisation between the piezo actuator controller and the measurement machine. Assuming that the actuator motion pattern consists of 1 s wide groove and 4 s of break performed periodically, two grooves may appear in the data. Out of them, one will meet conditions regarding the required data length before and after the groove, and the second may meet this requirement or may not. This way, even without synchronisation between devices at least one valid measurement was always achieved. For each depth 2 series of 15 measurements were performed. Data were stored in binary format inside Taylor Hobson ’.SBF’ files, represented as double-precision floating-point numbers. This resulted in 30 files per depth, 180 files in total.

### 2.4. Data Processing

**Figure 7:**
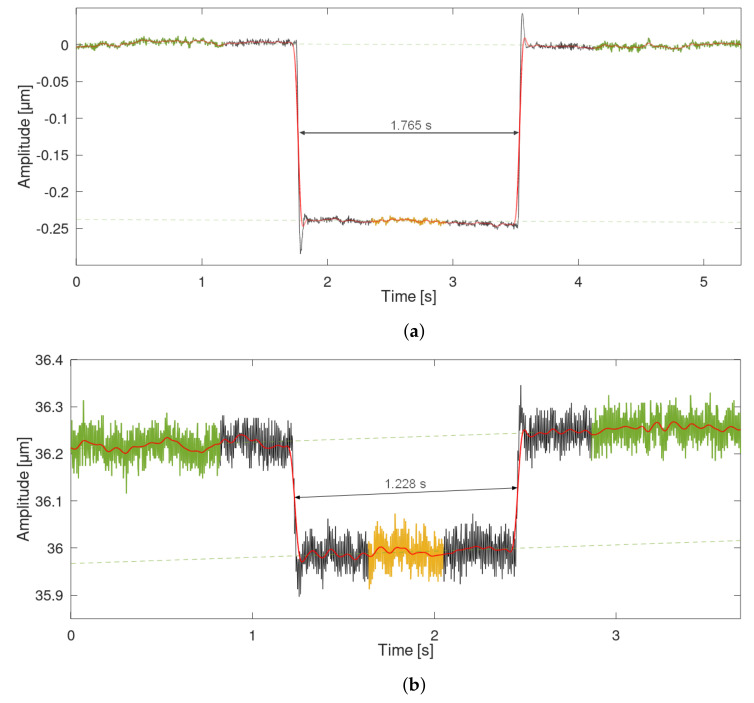
An example of 0.24 μm groove acquired with: (**a**) XL-80 laser interferometer; (**b**) Taylor Hobson 130 roundness measurement machine. The red line represents the profile after filtration with a Gaussian filter (cut-off length equal to 0.1 nominal width of the groove). Green-shaded areas are placed over external measurement lengths and the orange-shaded area is placed over the central measurement length. The green dashed lines represent the model fitted according to ISO 5436-1:2000 standard. These lines are extrapolated along the time axis for visual guidance. The nominal groove widths were 1.5 s and 1 s for the interferometric and roundness instrument measurements, respectively. The actual groove widths are marked on the graphs. The groove widths and consequently the size of measurement lengths were not affected by the filtration.

All the data processing was performed with Octave (GNU general public license). Prior to further processing, data obtained from the XL-80 interferometer were down-sampled 10-fold. Both, data from XL-80 interferometer and Taylor Hobson 130 roundness measurement machine were filtered with a Gaussian filter, using a cut-off length equal to 0.1 nominal width of the groove. The effect of filtration is observed in Figure 7.

#### 2.4.1. Estimation of Grooves’ Parameters

According to the ISO 5436-1:2000 [32] the step height of the real artefact should be calculated as follows:

Z=α×X+β+h×δ

where *Z* is a regression equation, *X* is a coordinate of the considered groove and the coefficients α, β, δ are being fitted with the least-squares method to the measurement lengths of a given profile (Figure 6). δ takes the value of +1 in the area of A and B and −1 in the area of C [32]. The depth of the groove *d* is equal to the double value of *h*.

The entire process of estimation of grooves’ parameters consisted of two steps. The first step involved an edge detection and a rough splitting of the data into sequences corresponding to separate grooves with an additional margin for subsequent calculations. In the second step, grooves’ parameters were estimated in an iterative way. Three iterations were performed. The initial groove’s width was based on the edge detection. During each iteration the measurement lengths were separated from the data, and the model was fitted according to ISO requirements, based on which the accurate groove depth was found. With the groove depth estimated, a new threshold for the edge detection was set and the procedure was repeated. After the third iteration, the estimation of groove’s parameters was supposed to be completed.

#### 2.4.2. Data Validation

After splitting into sequences corresponding to separate grooves and subsequent calculations, data obtained for each groove were validated. Data were excluded from the dataset under one of the three conditions: 1. The distance before or after the groove was too short, so ISO standard requirements [32] were not met; 2. The time span of the groove was shorter than 0.75 s (about 10 times the settling time of the piezo actuator response, 25% less than the assumed minimum acceptable width of the groove); 3. Mean values of amplitudes of measurement lengths prior to and after the groove differed more than 10 times the standard deviation of values within both measurement lengths, which might happen when the first piezo actuator movement was initialised while acquisition has already started.

#### 2.4.3. Overshot, Undershot and Settling Time Calculations

Data corresponding to separate grooves were subsequently used to monitor the signal behaviour near the edges of the groove. For each groove, the maximum signal undershot and overshot were found after the falling and the rising edge of the groove, respectively. After each edge, the settling time of the signal was also estimated. All these calculations were performed to ensure that the assumption regarding the minimum groove width required was consistent for the entire dataset.

## 3. Results

### 3.1. Calibration of the Piezo Actuator with the Laser Interferometer

**Figure 8:**
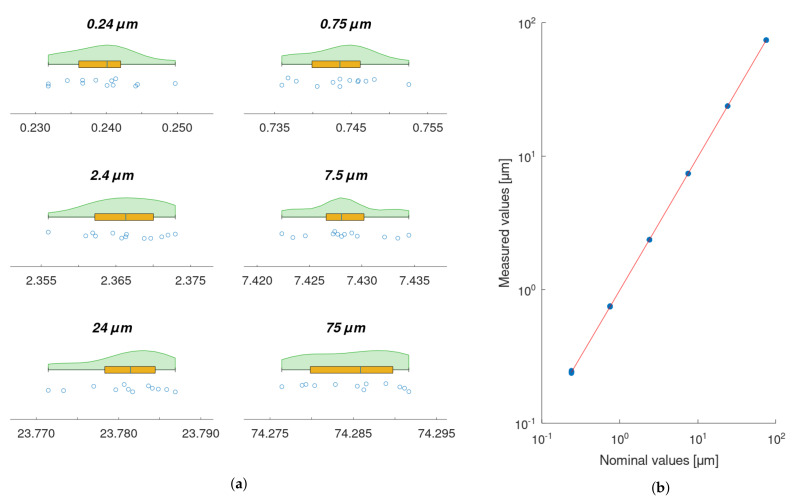
(**a**) Raincloud plots for each piezo actuator displacement being measured with XL-80 interferometer; (**b**) Scatterplot of data points representing the relationship between the required piezo actuator displacement and the displacement being measured with the XL-80 interferometer. The linear regression model is fitted ($1-R^{2}=6.4\times 10^{-8}$).

A least-squares linear regression was performed to model the relationship between the required position of the piezo actuator, set with the Kinesis software, and the actual displacements acquired with XL-80 interferometer ($1-R^{2}=6.4\times 10^{-8}$) —Figure 8b.

**Figure 9:**
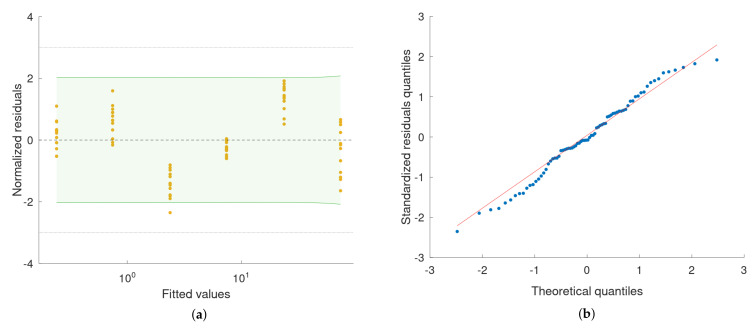
(**a**) Scatter plot of the normalised residuals (residuals divided by the standard deviation, σ=6.6 nm) vs. fitted values from the linear regression model. Shapiro–Wilk test for normality of residuals distribution: p=0.50. Dashed lines indicate the ±3σ zone. The green–shaded area represents the 95% prediction interval for a new observation based on the obtained model. (**b**) Quantile–quantile plot of the estimated residuals’ distribution against the normal distribution.

Data from the XL-80 interferometer, obtained for each displacement being measured, were characterised by means of raincloud plots (Figure 8a). Each plot includes three components: data points (a vertical jitter is introduced to help visual guiding of data); a boxplot with the lower whisker representing the smallest data point value within the 1.5 interquartile range below the third quartile and the upper whisker representing the largest data point value within the 1.5 interquartile range above the first quartile; Gaussian kernel density estimate smoothed according to Silverman’s rule. The quality of fitting was evaluated quantitatively by means of residuals analysis (Figure 9a). For the model, the prediction bounds in the analysed range (i.e., 0.24–75 μm) were calculated as well. These bounds are very narrow and would be difficult to visualise properly on the regression plot (Figure 8b), as it is presented in log-log scale. Thus, prediction bounds were presented on the residuals plot (Figure 9a) in the form of residual values, to support the visual validation of the obtained model. The maximum deviation of the estimated prediction bounds from the model equals 13.8 nm. For the residuals, the Shapiro–Wilk test for normality was calculated (p=0.50). A quantile–quantile plot is presented to support a visual assessment of the normality of the estimated residuals’ distribution. Sample (estimated residuals) quantiles are plotted against the normal distribution quantiles (Figure 9b).

### 3.2. Uncertainty Budget for Interferometer Measurement of the Piezo Actuator

The estimated parameters taken into account in the budget were gathered in Table 1. These are typical estimations of parameters adapted to this study. They do encompass most of the factors. However, in some cases, there are more internal parameters that are included within one to simplify the table, e.g., thermal factors.

Based on the data from Table 1 the combined uncertainty may be calculated as two times the square root of squares of each quantity multiplied by its sensitivity coefficients. The sensitivity coefficients of all the quantities in Table 1 are equal to 1, except for the Laser wavelength in the air for which the coefficient is 1/λ and the Index of refraction where the coefficient is multifactorial and takes into account components of the Edlen equation [33]. As calculated, the combined standard uncertainty was in the range from 17 nm for the groove of 7.5 μm to 19.0 nm for the groove of 75 μm, depending on the standard deviation taken into consideration for each step. Standard deviations were very consistent and they differed from each other only by a few nm, however, still being one of the main components of variability in such estimated uncertainty.

### 3.3. Calibration of the Roundness Measurement Machine with the Piezo Actuator

**Figure 10:**
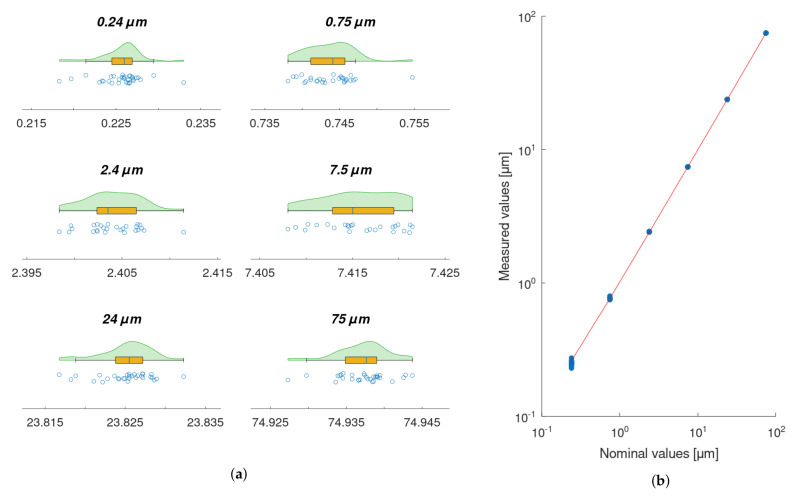
(**a**) Raincloud plots for each piezo-actuator displacement being measured with Taylor Hobson 130; (**b**) Scatterplot of data points representing the relationship between the piezo actuator displacement (estimated based on the required displacement and the calibration linear model obtained with the XL-80 interferometer) and the displacement being measured with the Taylor Hobson 130. The quadratic regression model is fitted ($1-R^{2}=3.1\times 10^{-7}$).

**Figure 11:**
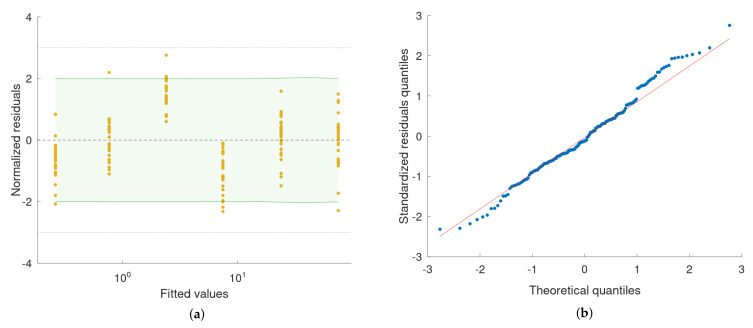
(**a**) Scatter plot of the normalised residuals (residuals divided by the standard deviation, σ=15.1 nm) vs. fitted values from the quadratic regression model. Shapiro–Wilk test for normality of residuals distribution: p=0.18. Dashed lines indicate the ±3σ zone. The green–shaded area represents the 95% prediction interval for a new observation based on the obtained model. (**b**) Quantile–quantile plot of the estimated residuals’ distribution against the normal distribution.

**Figure 12:**
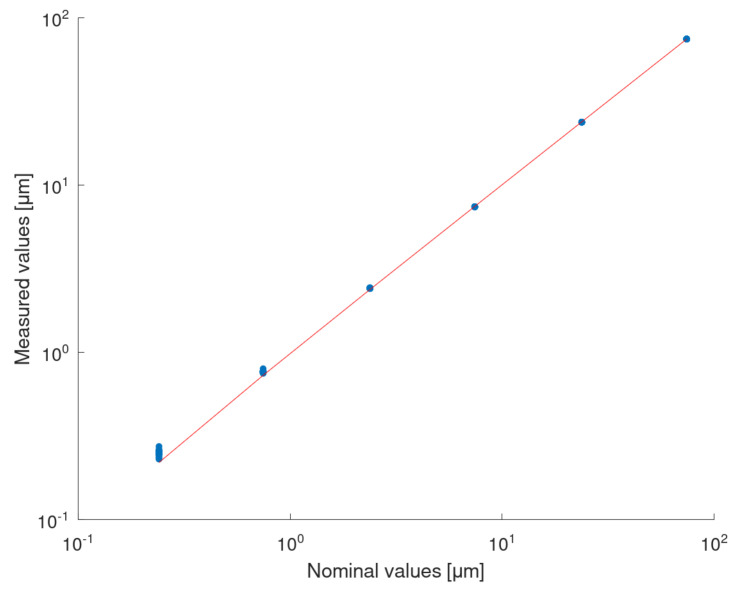
Scatterplot of data points representing the relationship between the piezo actuator displacement (estimated based on the required displacement and the calibration linear model obtained with the XL-80 interferometer) and the displacement being measured with the Taylor Hobson 130. The linear regression model is fitted ($1-R^{2}=4.9\times 10^{-6}$), slope: 1.008956, intercept: −0.023186μm.

**Figure 13:**
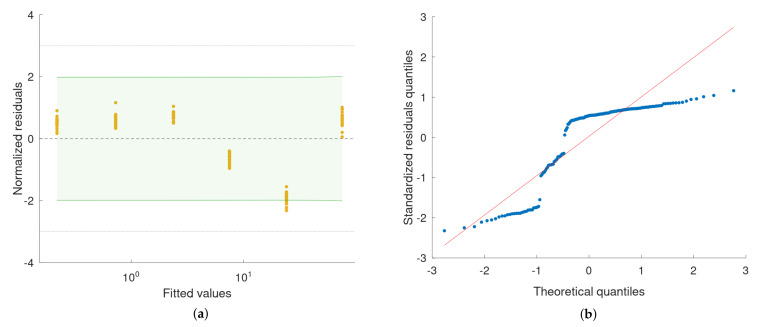
(**a**) Scatter plot of the normalised residuals (residuals divided by the standard deviation, σ=60.2 nm) vs. fitted values from the linear regression model. Shapiro–Wilk test for normality of residuals distribution: $p=1.76\times 10^{-15}$ indicates this distribution should not be assessed as normal. Deviations of the data points from the model might be interpreted as a graphical representation of the probe non-linearity. Dashed lines indicate the ±3σ zone. The green–shaded area represents the 95% prediction interval for a new observation based on the obtained model. (**b**) Quantile–quantile plot of the estimated residuals’ distribution against the normal distribution. The visible deviation of the sample distribution from normality confirms the low value of the Shapiro–Wilk test.

The model, obtained as a result of the piezo actuator calibration with the XL-80 interferometer, was used to correct the values of the piezo actuator displacement while performing the calibration of the Taylor Hobson 130 roundness measurement machine with the piezo actuator. The measurement data obtained with the Taylor Hobson 130 are presented in a form of raincloud plots (Figure 10a). A least-squares polynomial regression was used to fit a quadratic function to the calibration data (Figure 10b). The results of the fitting are summarised with residual and quantile–quantile plots (Figure 11a,b). A linear model was fitted to the data as well (Figure 12) and results are summarised in Figure 13a,b for residual and quantile–quantile plots respectively. Regarding data being considered, the linear model seems not to be a good choice. The quadratic estimation resulted in improved fitting outcomes: $1-R^{2}=3.1\times 10^{-7}$. The distribution of residuals might be assumed to follow the normal distribution (Shapiro–Wilk *p* = 0.18), 95% prediction bounds are at the level of 30.7 nm worst-case scenario.

### 3.4. Uncertainty Budget for Roundness Instrument

The typical budget for a roundness instrument was adapted for the piezo actuator measurements (Table 2). To estimate the uncertainties of influencing factors the uncertainties from the piezo actuator calibration (Section 3.2) were taken into account.

The estimated combined uncertainty for the roundness instrument would be, depending on the depth of the measured groove, ranging from 36 nm to 40 nm. The results are consistent and mostly depend on the uncertainty of the calibrated groove with the interferometer.

## 4. Discussion

It may be observed that, at given values, the displacement of the piezo actuator is highly repeatable and when calibrated with the interferometer the obtained uncertainty is below 20 nm worst-case scenario. Thus, it seems to be possible to reproduce the displacements to serve as a reference standard during one-, two- or multi-point calibration procedure of the roundness measurement machine.

A well-fitted linear model, which covers a relatively wide range of values, was also shown to be possible to obtain for the piezo actuator calibration data. Such a model provides an additional generalisation of the device behaviour and might provide some improvement to the calibration process. However, when using the calibration values estimated based on the regression model and not directly measured with the interferometer, the 95% prediction interval should be included in the overall calibration budget, to cover the additional uncertainty associated with the estimation. In the example calibration of the piezo actuator, given in the text, the prediction bounds are distant from the model by 13.8 nm (for 75 μm displacement) at worst-case and 13.3 nm (for 17.1 μm) at best-case scenario, while the double standard deviation is 10.4 nm (for 75 μm displacement) at worst-case and 2.6 nm (for 7.5 μm displacement) at best-case scenario.

The piezo actuator may be easily calibrated in many points along the measurement range under consideration, facilitating a multi-point calibration of the roundness measurement machine. The multi-point calibration may be used to estimate a linear or higher-order model. From a linear model, an accurate value of the slope can be derived to serve as a single value correction factor, which is often the case during the calibration of the roundness measurement machines. The deviations of the residuals, as those visible in Figure 13a may be interpreted as an internal nonlinearity of the device being analysed. It may happen, as in the example being shown, that these deviations are quite significant (95% prediction interval for a new observation results in the maximum predicted residual at the level of 120 nm). In such a case one- or two-point calibration, even for two different ranges, will result in rather a moderate correction for certain parts of the device measurement range. A higher-level model, such as the quadratic function in the given example, provides a much better estimation (Figure 11). With a piezo actuator, the transition from one- or two-point calibration to multi-point calibration might be done easily, as the procedure has a potential to be fully automatised.

### 4.1. Limitations of the Method

The precision and repeatability of a piezo actuator displacement are highly dependent on the stability and quality of the power supply and on the driver being used. If the piezo actuator is thought to serve as a standard, all the components required to perform the piezo actuator displacement should be a part of such a standard. The calibration of such a standard should be considered valid only for the given, exact configuration of devices.

The piezo actuator used during measurements presented in this study, controlled via its native software, did not hold time-based parameters properly, leading to the unnecessarily complicated data processing and validation. Software dedicated to dynamic and, at the same time, hi-precision measurements seems to be necessary to be developed. While applying a square excitation to the piezo actuator, ringing oscillations are present when the target position is reached. This enforces the use of a relatively long duration of the generated artefacts. By applying a fine software-based control of the piezo actuator deceleration this effect may be diminished, allowing to shorten the generated grooves, speeding up measurements, and acquiring more data to be analysed in the same amount of time, if needed. The data processing aimed to calculate the depth of grooves, necessary to obtain calibration curves for the roundness instrument, cannot rely, at the moment, on a typical roundness instrument software. Such software usually draws in the circle of reference to the data and then calculates, the parameter of RONt value (Roundness Total—ISO 12181) that would take the ringing oscillation into account and result in adding the peak oscillations amplitude to the groove size. Thus, such an approach forces the user to perform additional operations during measurements with roundness instruments. It is either special collecting of the data and then calculating the groove value rather than basing on algorithms within the device for calculating RONt or using the procedure of calibration if the instrument does have this additional procedure and enables to perform the testing in two separate static positions (not dynamic mode as in this study). Here, in this work, we use dynamic mode to gather more data and to statistically evaluate the feasibility of the new calibration route. In routine testing procedures of the device usually, not as many data are needed and so the one-time measurement of the portable gauge, as with gauge blocks, might be sufficient.

### 4.2. Prospects of the Method

It is worth mentioning that the limited time of measurement (40 s) did enhance largely the estimated uncertainty of the interferometric calibration. Thus, one might foresee calibration procedures under the more real working condition, where the piezo actuator is being characterised and calibrated within a longer time span and possibly with random sequences of artefact depths being considered. The performance of the system under various environmental conditions should also be verified. As a portable device for calibrations, it will be exposed to different conditions than in the laboratory when being transferred to the touching probe instruments to serve as the reference standard. Alternatively, integrating simultaneous calibration of the piezo actuator and the stylus probe [18,23,27] might be considered. Such a system, however, would not be as cost-effective as the presented solution. The sole calibration procedure using a piezo actuator proved to be effective and the estimated uncertainty for chosen grooves was of a similar order of magnitude as those obtained using gauge blocks. With gauge blocks it is impossible to perform dynamic procedures, hence the data are being collected using the static calibration option. The wrung gauge block is being moved under the probe to estimate the height of the step. A single measurement is sufficient for the roundness instrument software for necessary calibration but it is burdened with errors. Thus, the procedure is repeated. However, it is impossible for the user to put the gauge blocks always in the same position. Thus, additional factor plays a role. With a piezo actuator, we are able to eliminate the lateral movement underneath the probe and to ensure the same position of the sensor in the repeatedly performed tests. In this study, the repeatability of the tests was consistent, with a standard deviation of several to dozen nm. So in comparison with the gauge blocks procedure, where a standard deviation might be in hundreds of nm due to the above-mentioned factors, the piezo actuator seems to be a reasonable alternative. Moreover, it is free from the wringing operation which is also strongly user-dependent. The only considerations, when applying piezo actuator in calibrator procedures, which must be taken into account are the piezo actuator itself and its characterisation.

## 5. Conclusions

Thanks to the flexibility of the method the user is able to generate any chosen step in a given piezo actuator range. It is very convenient, taking into account that certain measuring tasks will have roundness data around single μm (RONt) while others could be of higher value. Focusing solely on calibrating or adjusting the gain factor on the step built with gauge blocks or flick standard, could limit the knowledge of the probe’s behaviour to values being used during the calibration, not giving any information on the behaviour further away from the value of the step. Thus multi-step method or choosing an appropriate step for a specific purpose should be of greater value for the user.

## Figures and Tables

**Table 1:** Typical budget for calculating the uncertainty of interferometric measurements adapted for the considered study.

Quantity	Uncertainty Contribution nm
Repeatability	for each groove nm *
Interferometer	0.3
Piezo actuator	1.7
Laser wavelength in air	0.1
Index of refraction	negligible
Interferometer nonlinearity	2.3
Thermal factors	0.1
Dead-path error	5.1
Abbé error	5.8
Cosine error	0.1

* The standard deviation for the respective grooves 0.24 µm (3.1 nm); 0.74 µm (3.3 nm); 2.4 µm (3.0 nm); 7.5 µm (1.3 nm); 24 µm (2.9 nm); 75 µm (5.2 nm).

**Table 2:** Typical budget for calculating the uncertainty of roundness measurements adapted for the considered study of roundness instrument.

Quantity	Uncertainty Contribution in nm
Instrument resolution	4
Uncertainty of determining the depth of the groove (from simulated grooves)	17 to 19
Groove measurement repeatability	for each groove nm *

* For each simulated groove the standard deviation were 0.24 µm (8.8 nm); 0.74 µm (9.6 nm); 2.4 µm (8.0 nm); 7.5 µm (14.0 nm); 24 µm (10.2 nm); 75 µm (12.2 nm).

## Data Availability

The data presented in this study are openly available in Zenodo at [https://doi.org/10.5281/zenodo.7297897].
